# Editorial: Immunity to Emerging Pathogens in Poikilothermic Vertebrates

**DOI:** 10.3389/fimmu.2022.867818

**Published:** 2022-03-15

**Authors:** Eva-Stina Edholm, Leon Grayfer

**Affiliations:** ^1^ Norwegian College of Fishery Science, Faculty of Biosciences, Fisheries & Economics, University of Tromsø-The Arctic University of Norway, Tromsø, Norway; ^2^ Department of Biological Sciences, George Washington University, Washington, DC, United States

**Keywords:** editorial, emerging pathogens, poikilothermic vertebrates, fish, amphibian

Our world is changing. Global warming and anthropogenic activities are culminating in devastating consequences to aquatic habitats and to the numerous cold-blooded vertebrate species that inhabit them. Together with overcrowding in aquaculture settings, these changes are seeing the emergence of new and opportunistic pathogens that are decimating wild and cultured aquatic animal populations of fish and amphibians. With these growing environmental and economic concerns, we are charged with developing measure through which to counteract and prevent such infection outbreaks. However, this requires further advances in our current understanding of the immunological successes and shortcomings of these poikilothermic vertebrates when dealing with infectious agents. In general, aquatic vertebrates such as bony fish and amphibians possess many hallmarks associated with mammalian immunity but have also adopted some unique strategies for immune cell development and antimicrobial defenses. Arguably, these animals possess much less proficient adaptive immune systems and must thus rely more heavily on their innate immune defenses for controlling infectious agents. Indeed, the immune systems of poikilothermic vertebrates have already been shown to possess several key differences from terrestrial mammals. These include but are not limited to distinct repertoires of key cytokines and cell signaling components, disparate leukocyte differentiation pathways and unique pathogen recognition strategies.

This special issue of Frontier of Immunology coalesces the recent advances in the understanding of aquatic vertebrates, and specifically bony fish and amphibian immune defenses, the emerging pathogens that plague them and the interfaces between these pathogens and their cold-blooded host immune systems. This special issue serves as a dedicated overview of recent progress in research areas related to teleost fish and amphibian host innate immune responses and host-pathogen interactions. Through this collection of 8 articles, including both original research as well as a comprehensive review, we coalesce recent advances in the current understanding of the innate immune responses of aquatic vertebrates.

## Fish Aquaculture and Immunity

Increased research into aquatic animal health and immunity has been motivated in large part by the growing needs of aquaculture industries. Rearing animals like fish in relatively restrictive and high-density habitats renders them more prone to opportunistic and epizootic infections. As such, developing a greater understanding of which fish immune components may be compromised under such stress and/or disease scenarios concurrently grant greater insights into fish immune responses and may present potential avenues by which to develop novel therapeutic approaches and better rearing practices.

The Gram-negative bacteria, *Flavobacterium psychrophilum* is the causative agent of Cold Water Disease that results in devastating losses to farmed salmonid fish. The limited understanding of the aquaculture conditions that manifest in such disease outbreaks has precluded the development of adequate preventative measures against this disease. By examining the effects of recirculated water on genetically distinct lines of rainbow trout, Everson et al. demonstrated that *F. psychrophilum-*infected fish mortalities differed between genetic lines and were more extensive for animals reared in reused water. Interestingly, fish vaccinated for the Infectious Hematopoietic Necrosis Virus (IHNV) not only had lower IHNV loads but also possessed lower *F. psychrophilum* loads. Moreover, while a large proportion of fish presented with combined IHNV and *F. psychrophilum*, the vaccine-mediated protection was lost in fish experimentally coinfected with IHNV and *F. psychrophilum*. Together this underlines the complex interactions between fish environmental conditions, animal immunological histories and possible co-infections under farm and undoubtedly wild conditions.

The Gram-positive intracellular bacteria, *Renibacterium salmoninarum* is the causative agent of Bacterial Kidney Disease (BKD). In turn, Lumpfish are commonly used as “cleaner fish” to biocontrol sea lice on Atlantic salmon stocks and in a manuscript included in this special issue, Gnanagobal et al. explored the susceptibility and by extension, the possible role of Lumpfish as vectors of *R. salmoninarum.* The *R. salmoninarum*-infected Lumpfish exhibited characteristic BKD and the bacteria persisted within the heavily infected fish up to 98 days post infection, suggesting that indeed *R. salmoninarum*-infected Lumpfish may serve as reservoirs and vectors of this pathogen. Moreover, while the lumpfish innate immune responses were elevated following infection, *R. salmoninarum*-infected Lumpfish displayed depressed expression of immune components associated with adaptive immunity. More work of this nature is needed to discern how and under what contexts Lumpfish are infected, harbor and transmit *R. salmoninarum* to farmed salmon.

## Transcriptomics-Mediated Characterization of Fish Host-Pathogen Interactions

Aquatic vertebrate species present intriguing alternative animal models by way of which to explore the evolutionary bases of convergent and diverged host-pathogen interactions. Investigations of, and using such organisms impose limitations due to the general lack of available species-specific resources. Fortunately, the development and accessibility of several transcriptomic approaches have circumvented many of such limitations and have permitted researchers to delve deeper into the interactions between pathogens and their respective aquatic animal hosts. This special issue includes two such studies.

The Gram-negative intracellular bacteria, *Yersinia ruckeri* is the causative agent of fish enteric red mouth disease. This pathogen exhibits a broad host range and results in significant economic losses to aquaculture industries around the globe. Using transcriptomic analyses of *Y. ruckeri*-infected Channel catfish trunk kidneys, Yang et al. underlined the dynamics of these pathogen-fish host interactions, thus defining the roles of distinct cytokine, immune receptor and cell signaling networks in these processes.

The Gram-negative intracellular bacteria, *Piscirickettsia salmonis* results in piscirickettsiosis disease, which is one of the most devastating diseases of salmonid fish and a major detriment to aquaculture. Through time-series analyses of Atlantic salmon infected with *P. salmonis*, Xue et al. separated infected animals into modestly and substantially infected groups. Using microarray analyses, Xue et al. then compared the transcriptional profiles of the heavily- and modestly infected animals to discern possible immune markers of fish susceptibility and resistance to P. salmonis.

With more studies like the ones above, which take big-picture approaches to intracellular bacterial infections of fishes, we can begin to draw parallels and contrasts across fish and pathogen-specific interactions, thereby filling in the gaps in our understanding of both fish immunity in general as well disparate fish host-pathogen interactions.

## Fish Macrophage-Mediated Responses

Macrophage-lineage cells are indispensable to the biology and immunity of all vertebrates examined to date. These cells are key to recognizing infiltrating pathogens, coordinating the ensuing immune responses and often bridging the innate and adaptive immune arms. In this special issue, we feature two primary articles that describe macrophage biology in the context of fish infectious diseases.

Piscine Orthoreovirus (PRV) is a segmented double-stranded RNA virus belonging to the *Orthoreovirus* genus, which causes cardiac and muscle inflammation in farmed Atlantic salmon, with an apparent tropism for fish macrophages. In turn, macrophage functionality is often described with respect to a spectrum between the M1 inflammatory (classically activated) and M2 healing/resolving (alternatively activated) polarization states. Malik et al. examined the M1/M2 polarization states of the afflicted salmon macrophages in the context of experimentally PRV-1-induced heart and skeletal muscle inflammation. Their results suggest that PRV-1 localized within M1-polarized macrophages in both the fish heart and skeletal muscle tissues. Conversely, while M2-polarized macrophages were broadly distributed within these tissues, they did not appear to contain the virus. Moreover, the fish immune responses to the PRV-1 infection were marked by CD8+ cell infiltration and increased MHC class I expression within the heart tissues and less so in the infected fish skeletal muscle tissues. It will be interesting to see with further studies how fish macrophage functional polarization dictates susceptibility and resistance to viral as well as other important fish pathogens.

Presumably because macrophages tend to be long-lived, terminally differentiated and play integral roles in animal physiologies and defenses, they are often also used as vectors of intracellular pathogen persistence and dissemination. On this note, pathogenic mycobacteria species thrive by infecting the macrophages of their respective hosts and examining the interactions between *M. fortuitum* and catfish kidney-derived macrophages, Dahiya et al. demonstrated that the infected macrophage activation of toll like receptor 2 culminates in mitochondrial stress, resulting in mitochondrial reactive oxygen species (ROS) production, which is an important mechanism for eliminating the invading mycobacteria.

## Amphibian Antiviral Responses

The global amphibian biodiversity is threatened by members of the *Ranavirus* genus (family *Iridoviridae*), emphasizing the need to gain greater insights into the ranavirus-amphibian host interactions and the antiviral responses mounted by distinct amphibian species against such pathogen. One of the articles in this special issue coalesces the current understanding of a *Ranavirus* genus member and another showcase findings pertaining to amphibian host-ranavirus interactions.

The Chinese Giant Salamander Iridovirus (GSIV) ranavirus is an important pathogen to Chinese aquaculture and economy. While a large proportion of ranavirus-amphibian host interactions has focused on other model organisms, in a review article by Jiang et al., the authors underline and highlight the specific immune system and antiviral responses of the Chinese giant salamander as well as proposing effective management measures for GSIV in giant salamander farming practices.

While it is apparent that the interactions between ranaviruses and their tadpole and post-metamorphic amphibian hosts are distinct, the specifics of these differences remain enigmatic. In an article by Hauser et al., the authors show that myeloid lineage cells are recruited to the intestines of Frog Virus 3 (FV3) ranavirus-infected *Xenopus laevis* tadpoles, wherein these cells facilitate antiviral interferon responses. Conversely, such cells are resident within the intestines of post-metamorphic *X. laevis*, possibly contributing to their resistance against FV3 infections.

## Concluding Remarks

The primary articles and review featured in this special issue are just some examples of the exciting new foyers into innate immunity of aquatic vertebrates. As the field of comparative immunology grows with such research, we are granted clearer windows into the unique and intriguing strategies by which these animals orchestrate their antimicrobial defenses. Indeed, studies and articles like those featured here, will be instrumental to the development of better aquacultural practices, aquatic habitat preservation and remediation and will lead to more complete understanding of the evolution of vertebrate immune responses.

## Author Contributions

All authors listed have made a substantial, direct and intellectual contribution to the work, and approved it for publication.

## Funding

LG acknowledges support from NSF (IOS: 1749427). E-SE acknowledges support from the Tromsø Research Foundation Starting Grant and by the Aquaculture program of The Research Council of Norway (Grant No. 295036).

## Conflict of Interest

The authors declare that the research was conducted in the absence of any commercial or financial relationships that could be construed as a potential conflict of interest.

## Publisher’s Note

All claims expressed in this article are solely those of the authors and do not necessarily represent those of their affiliated organizations, or those of the publisher, the editors and the reviewers. Any product that may be evaluated in this article, or claim that may be made by its manufacturer, is not guaranteed or endorsed by the publisher.

